# Supplementary material for: Physicochemical and Adsorption Characteristics of Divinylbenzene-co-Triethoxyvinylsilane Microspheres as Materials for the Removal of Organic Compounds
Source: Molecules. 2021 Apr 20;26(8):2396. doi: 10.3390/molecules26082396 (PMC8074589; doi:10.3390/molecules26082396)
Supplement: Supplementary file 1 [file molecules-26-02396-s001.zip › molecules-1145552-supplementary.pdf]

Supplementary material for the paper:

Physicochemical and Adsorption Characteristics of Divinylbenzene-*co*-Triethoxyvinylsilane Microspheres as Materials for the Removal of Organic Compounds

Alicja Bosacka*, Małgorzata Zienkiewicz-Strzalka, Małgorzata Wasilewska, Anna Derylo-Marczewska* and Beata Podkoscielna

Institute of Chemical Sciences, Faculty of Chemistry, Maria Curie-Sklodowska University, Maria Curie-Sklodowska Sq. 3, 20-031 Lublin, Poland;

malgorzata.zienkiewicz@poczta.umcs.lublin.pl (M.Z.-S.); malgorzata.wasilewska@poczta.umcs.lublin.pl (M.W.); beatapod@poczta.umcs.lublin.pl (B.P.);

* Correspondence: alicja.bosacka@poczta.umcs.lublin.pl (A.B.); annad@hektor.umcs.lublin.pl (A.D.-M.); Tel.: +48-81-53-777-66 (A.D.-M.);

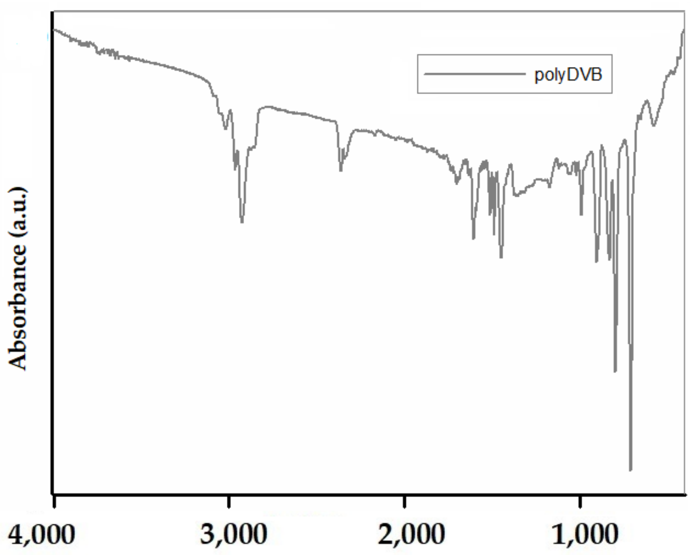

Figure S1. FTIR/ATR spectra of DVB.

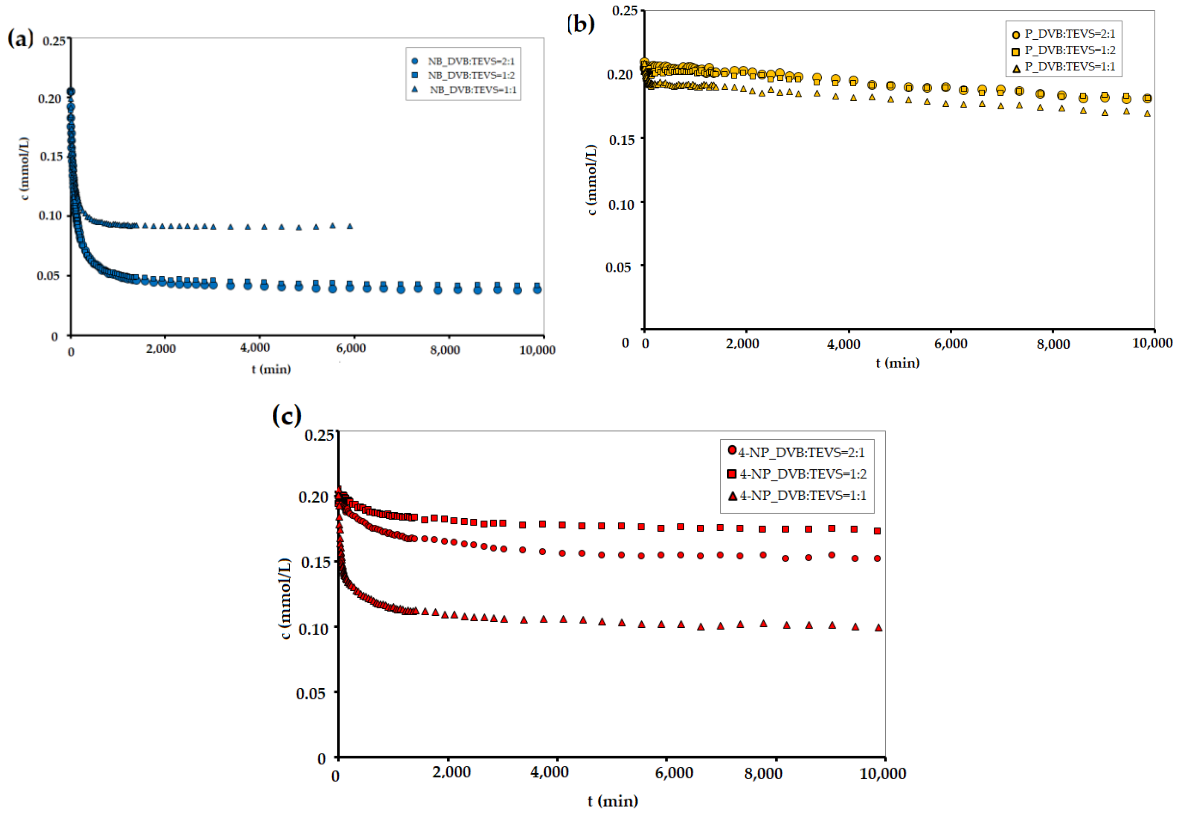

Figure S2. Adsorption kinetics for NB (**a**), P (**b**) and 4-NP (**c**) on DVB:TEVS=2:1, DVB:TEVS=1:2 and DVB:TEVS=1:1 presented as changes in concentration over time.

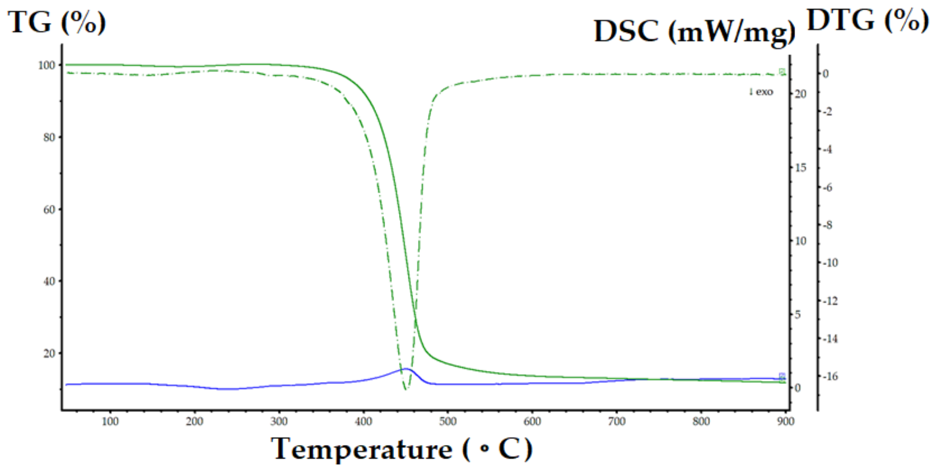

Figure S3. TG/DTG/DSC curves of DVB.

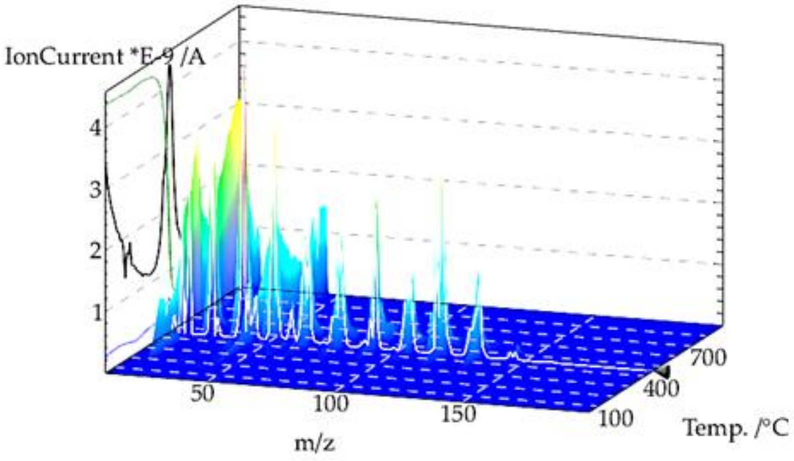

Figure S4. 3D MS profile of thermal decomposition of DVB.

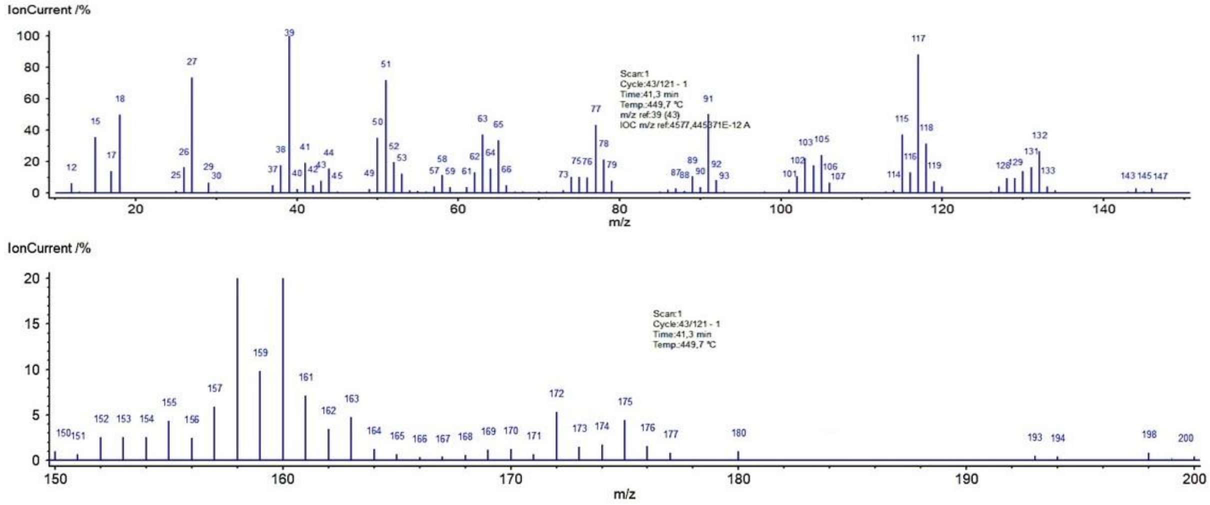

Figure S5. MS spectra of thermal degradation of DVB at 450 ◦C.

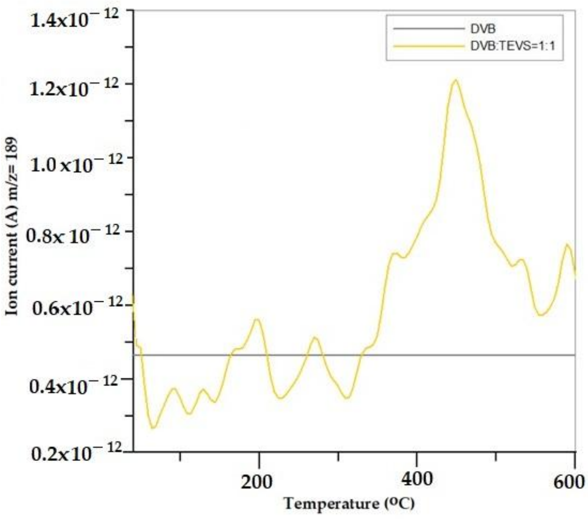

Figure S6. MS profiles of gaseous products of thermal degradation of DVB:TEVS=1:1 and DVB (m/z=189).
